# Inhibition of hepatic natural killer cell function via the TIGIT receptor in schistosomiasis-induced liver fibrosis

**DOI:** 10.1371/journal.ppat.1011242

**Published:** 2023-03-17

**Authors:** Yuan Gao, Xiaocheng Zhang, Tingting Jiang, Hao Zhou, Hua Liu, Yuan Hu, Jianping Cao

**Affiliations:** 1 National Institute of Parasitic Diseases, Chinese Center for Disease Control and Prevention (Chinese Center for Tropical Diseases Research); Key Laboratory of Parasite and Vector Biology, National Health Commission of People’s Republic of China; World Health Organization Collaborating Center for Tropical Diseases, Shanghai, China; 2 The School of Global Health, Chinese Center for Tropical Diseases Research, Shanghai Jiao Tong University School of Medicine, Shanghai, China; George Washington University, UNITED STATES

## Abstract

Schistosomiasis is a zoonotic parasitic disease. *Schistosoma japonicum* eggs deposited in the liver tissue induce egg granuloma formation and liver fibrosis, seriously threatening human health. Natural killer (NK) cells kill activated hepatic stellate cells (HSCs) or induce HSC apoptosis and inhibit the progression of liver fibrosis. However, the function of NK cells in liver fibrosis caused by *S*. *japonicum* infection is significantly inhibited. The mechanism of this inhibition remains unclear. Twenty mice were percutaneously infected with *S*. *japonicum* cercariae. Before infection and 2, 4, 6, and 8 weeks after infection, five mice were euthanized and dissected at each time point. Hepatic NK cells were isolated and transcriptome sequenced. The sequencing results showed that *Tigit* expression was high at 4–6 weeks post infection. This phenomenon was verified by reverse transcription quantitative PCR (RT-qPCR) and flow cytometry. NK cells derived from *Tigit*^-/-^ and wild-type (WT) mice were co-cultured with HSCs. It was found that *Tigit*^-/-^ NK cells induced apoptosis in a higher proportion of HSCs than WT NK cells. Schistosomiasis infection models of *Tigit*^-/-^ and WT mice were established. The proportion and killing activity of hepatic NK cells were significantly higher in *Tigit*^-/-^ mice than in WT mice. The degree of liver fibrosis in *Tigit*^-/-^ mice was significantly lower than that in WT mice. NK cells were isolated from *Tigit*^-/-^ and WT mice and injected via the tail vein into WT mice infected with *S*. *japonicum*. The degree of liver fibrosis in mice that received NK cell infusion reduced significantly, but there was no significant difference between mice that received NK cells from *Tigit*^-/-^ and WT mice, respectively. Our findings indicate that *Tigit* knockout enhanced the function of NK cells and reduced the degree of liver fibrosis in schistosomiasis, thus providing a novel strategy for treating hepatic fibrosis induced by schistosomiasis.

## Author summary

Schistosomiasis is a serious zoonotic parasitic disease. *Schistosoma japonicum* egg deposition in the liver induces granuloma formation and liver fibrosis, which are the main causes of death in patients with chronic and advanced schistosomiasis. Identifying the regulatory mechanism of liver fibrosis in schistosomiasis is crucial to inhibiting its progression. Natural killer (NK) cells kill activated hepatic stellate cells or induce their apoptosis and stop the progression of liver fibrosis. We found that TIGIT, an inhibitory receptor, was significantly highly expressed on NK cells at 4 and 6 weeks post infection, which may be associated with NK cell inhibition in schistosomiasis. We further explored the role of the TIGIT receptor in regulating NK cell-killing activity and liver fibrosis in schistosomiasis. Results indicate that blocking TIGIT receptors activated NK cells and decreased the extent of liver fibrosis, providing a novel target for treating liver fibrosis in schistosomiasis.

## Introduction

Schistosomiasis is a zoonotic parasitic disease caused by *Schistosoma japonicum*, mainly in tropical and subtropical regions [1]. According to the National Schistosomiasis Epidemic Status 2020, the prevalence of schistosomiasis in China has reduced. However, many patients with schistosomiasis exhibit advanced liver fibrosis [2]. After *S*. *japonicum* infection, *S*. *japonicum* eggs deposited in the host liver release soluble egg antigens, which induce the host immune response and activate hepatic stellate cells (HSCs) to transform into myofibroblasts, and secrete a high amount of extracellular matrix [3,4]. The excessive deposition of extracellular matrix in the liver leads to liver fibrosis.

Natural killer (NK) cells, as part of the innate immune system, are the first line of defense against acute infections and regulate adaptive immune responses [5,6]. NK cells constitute a major subset of hepatic non-parenchymal cells, accounting for 10%–15% of hepatic lymphocytes in mice and 30%–50% in humans and rats [7,8]. A previous study reports that NK cells killed activated HSCs by producing interferon (IFN)-γ, interacted with RAE-1 through NKG2D to destroy HSCs, or induced their apoptosis, thus inhibiting liver fibrosis [9]. NK cells have also been reported to inhibit liver fibrosis caused by the hepatitis virus [10]. Earlier studies have shown that *S*. *japonicum* infection-induced activation of NK cells produced IFN-γ and killed early-activated HSCs. In contrast, another study reported that liver fibrosis was significantly aggravated when NK cells were depleted [11].

The cytotoxicity of NK cells is regulated by activating and inhibitory receptors [12]. Common NK cell activating receptors in mice include NKG2D, NKp46, NKp30, and NKG2C, and inhibitory receptors include TIGIT, NKG2A, and TIM3. When the target cells do not express major histocompatibility complex (MHC)-I molecules, or when specific ligands directly recognize the activating receptor, the inhibitory signal is diminished, the activating signal is enhanced, and NK cells exhibit killing activity [13]. Most inhibitory receptors on the surface of NK cells transmit inhibitory signals by recognizing MHC-I molecules, thereby inhibiting NK cell function and contributing to autoimmune tolerance to avoid killing normal cells [14].

Our previous study found that intrahepatic NK cells were activated approximately 2–4 weeks after infection with *S*. *japonicum*. However, NK cell function was inhibited 6 weeks post infection, as high numbers of eggs were deposited in the host liver [15]. However, the intrahepatic NK cell inhibition mechanism in the later stages of *S*. *japonicum* infection remains unclear. Therefore, we established a mouse model of *S*. *japonicum* infection, isolated hepatic NK cells, and used transcriptome sequencing to detect NK cell receptor expression changes. We screened and verified the receptors associated with NK function inhibition and explored the potential mechanisms of NK cell inhibition and liver fibrosis induced by *S*. *japonicum* infection.

## Results

### Transcriptome profiling of natural killer cells in schistosomiasis-induced liver fibrosis

**Fig 1:**
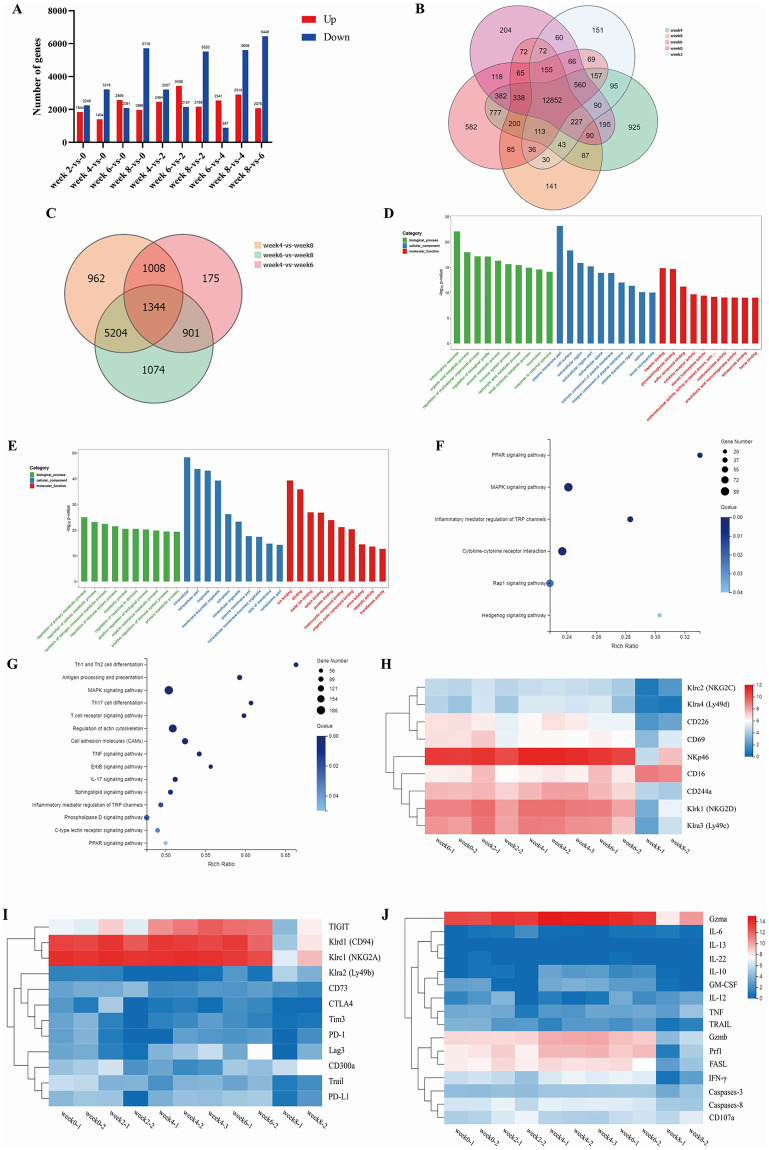
Analysis of natural killer (NK) cell transcriptome sequencing data. (A) Differentially expressed genes (DEGs) at different time points after infection. (B) Venn diagram showing DEG overlap between the two groups at 0, 2, 4, 6, and 8 weeks post infection. (C) Venn diagram showing DEG overlap between the two groups at the 4, 6, and 8 weeks post infection. (D) Bar graphs of gene ontology (GO) enrichment of DEGs common between 6 weeks and 4 weeks post infection. (E) Bar graphs of GO enrichment of DEGs common between 8 weeks and 6 weeks post infection. (F) Bubble plots of Kyoto Encyclopedia of Genes and Genomes (KEGG) pathway enrichment of DEGs common between 6 weeks and 4 weeks post infection. (G) Bubble plots of KEGG pathway enrichment of DEGs common between 8 weeks and 6 weeks post infection. (H) Expression of NK cell activating receptors at different time points post infection. (I) Expression of NK cell inhibitory receptors at different time points post infection. (J) Expression of NK cell functional molecules at different time points post infection.

The number of differentially expressed genes (DEGs) (Fig 1A) was higher at 8 weeks post infection than at 0, 2, 4, and 6 weeks post infection. At 6 weeks post infection, the number of upregulated genes was significantly higher than that of downregulated genes. However, the opposite trend was observed 8 weeks post infection (Fig 1A). A Venn diagram of the DEGs at each time point showed that the number of unique DEGs was the highest at 4 weeks post infection, followed by that at 6 weeks post infection (Fig 1B). The smallest overlap was observed between DEGs identified at 4 and 6 weeks post infection, and the highest overlap was observed between DEGs identified at 6 and 8 weeks post infection (Fig 1C).

Gene ontology (GO) analysis showed that the DEGs common between those identified at 6 and 4 weeks post infection were most significantly enriched in inflammatory responses, plasma membrane fraction, and heparin binding in the biological process, cellular component, and molecular function categories, respectively (Fig 1D). GO analysis of the DEGs common between those identified at 8 and 6 weeks post infection (Fig 1E) showed that they were most significantly enriched in regulation of primary metabolic processes, intracellular, and ion binding in the biological process, cellular component, and molecular function categories, respectively. The Kyoto Encyclopedia of Genes and Genomes (KEGG) pathway enrichment analysis showed that the DEGs common between those identified at 6 and 4 weeks post infection were associated with inflammation regulatory pathways such as the PPAR and MAPK signaling pathways (Fig 1F). The DEGs common between those identified at 8 and 6 weeks post infection were associated with inflammation pathways such as Th cell differentiation and MAPK signaling pathways (Fig 1G).

The expression levels of NK cell receptors were higher at 4 and 6 weeks post infection and were lower at 8 weeks post infection. Between 0 and 6 weeks post infection, the levels of activating receptors of NK cells were high (Fig 1H), whereas the levels of most inhibitory receptors were low (Fig 1I). The mRNA expression of *Tigit*, *CD94*, and *Nkg2A* significantly increased from 2 to 6 weeks post infection and decreased after 6 weeks post infection. The expression of *Tigit* showed the most marked changes.

The expression levels of NK cell functional molecules (Fig 1J), such as *Ifn-γ*, *perforin (Prf)1*, and *granzyme B (Gzmb)*, significantly decreased at 8 weeks post infection. The expression levels of *Gzma*, *Gzmb*, *Gzmk*, and *Prf1* were markedly lower at 6 weeks than at 4 weeks post infection, suggesting that NK cell function was inhibited from 6 to 8 weeks post infection. The expression levels of interleukin (*Il*)-10, GM-CSF, and FASL increased from 4 to 6 weeks post infection. In contrast, there were no significant differences in the expression levels of *Il-6*, *Il-13*, *Il-22*, *caspase-3*, and *caspase-8* from 0 to 8 weeks post infection.

### Changes in *Tigit* expression in mouse hepatic natural killer cells

**Fig 2:**
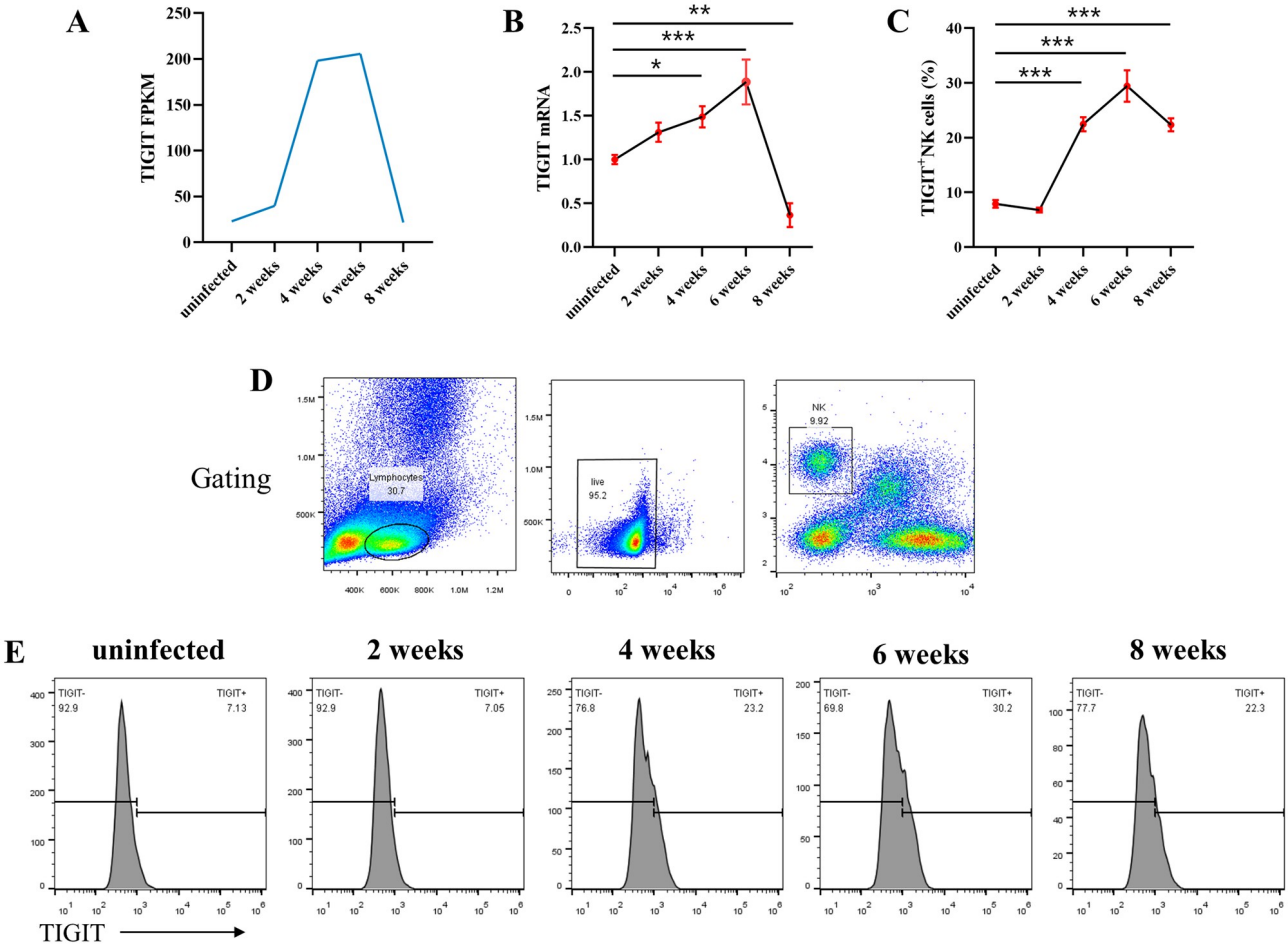
Changes in *Tigit* expression on hepatic natural killer (NK) cells of mice infected with *Schistosoma japonicum*. (A) Expression levels of *Tigit* in hepatic NK cells at different time points after *S*. *japonicum* infection (FPKM). (B) Relative mRNA transcript levels of *Tigit* in hepatic NK cells at different time points after *S*. *japonicum* infection. (C) Dynamic changes in the proportion of *Tigit*^+^ NK cells at different time points after *S*. *japonicum* infection. (D) Circle gating strategy of flow cytometry analysis of lymphocytes, viable cells, and NK cells among hepatic non-parenchymal cells. (E) Histogram of the proportion of *Tigit*^+^ NK cells in the liver at different time points after infection. All data are presented as mean ± standard deviation of at least three independent experiments. *P < 0.05, **P < 0.01, ***P < 0.001.

An analysis of the NK cell transcriptome sequencing data (Fig 2A) showed that the mRNA expression of the inhibitory receptor TIGIT on NK cells was significantly higher at 4 and 6 weeks post infection than that of other time points. Reverse transcription-quantitative PCR (RT-qPCR) and flow cytometry showed that the mRNA expression of *Tigit* significantly increased from week 4 to 6 post infection and significantly decreased from week 6 to 8 post infection. These findings were consistent with the transcriptome sequencing results (Fig 2B and 2C).

### *Tigit* knockout enhanced the killing activity of natural killer cells

**Fig 3:**
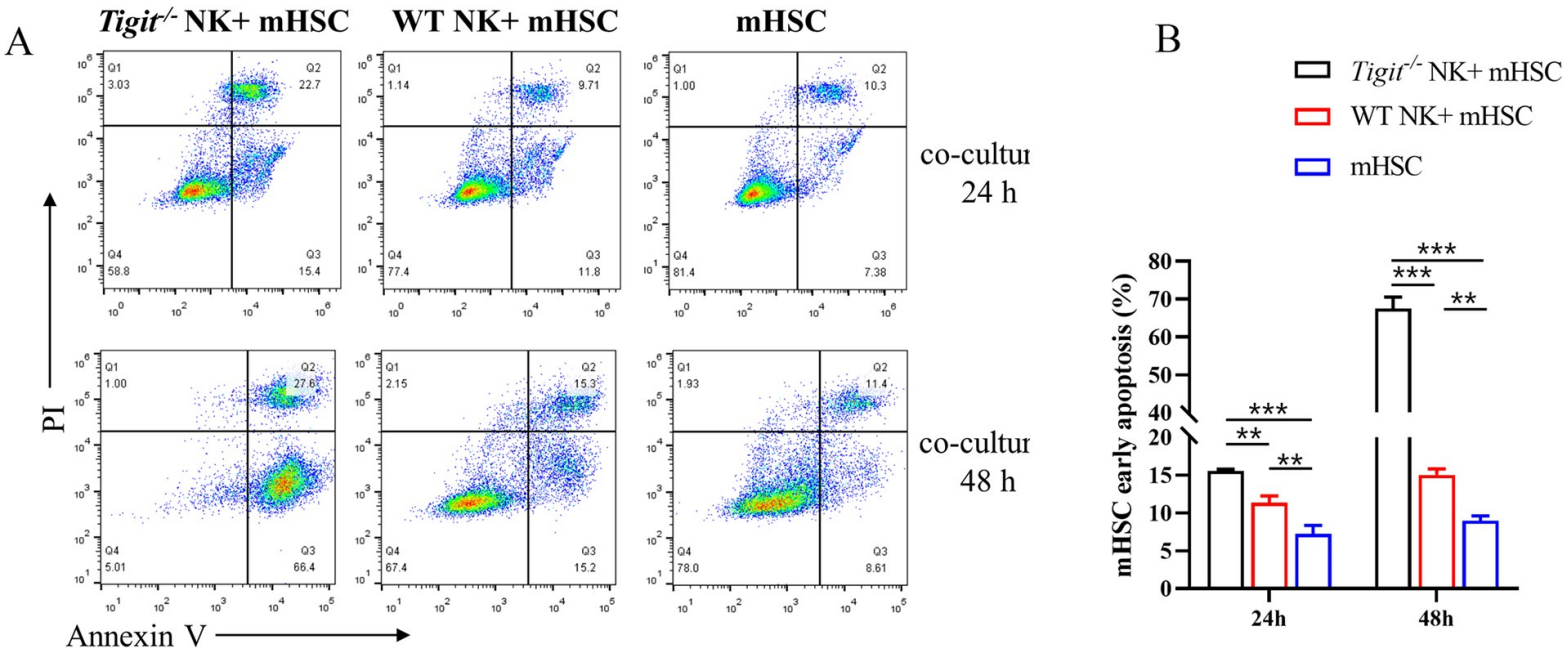
*Tigit* knockout enhanced natural killer (NK) cell killing activity on hepatic stellate cells (HSCs). (A) Flow cytometry scatter plots signifying mouse HSC (mHSC) apoptosis after co-culture with NK cells for 24 and 48 h. (B) Histogram of the percentage of apoptotic mHSCs after co-culture with NK cells for 24 and 48 h. All data are presented as mean ± standard deviation of at least three independent experiments. *P < 0.05, **P < 0.01, ***P < 0.001.

Hepatic NK cells derived from *Tigit*^*-/-*^ and WT mice were co-cultured with mouse HSCs (mHSCs) in vitro, and the apoptosis ratio of mHSCs was determined using flow cytometry. After 24 and 48 h of co-culture, the early apoptosis ratio of mHSCs co-cultured with NK cells was higher than that in the control group. The ratio of mHSCs co-cultured with the *Tigit*^*-/-*^ NK was significantly higher than that with WT NK (Fig 3). The results indicated that *Tigit* knockout enhanced the killing effect of NK cells on HSCs.

**Fig 4:**
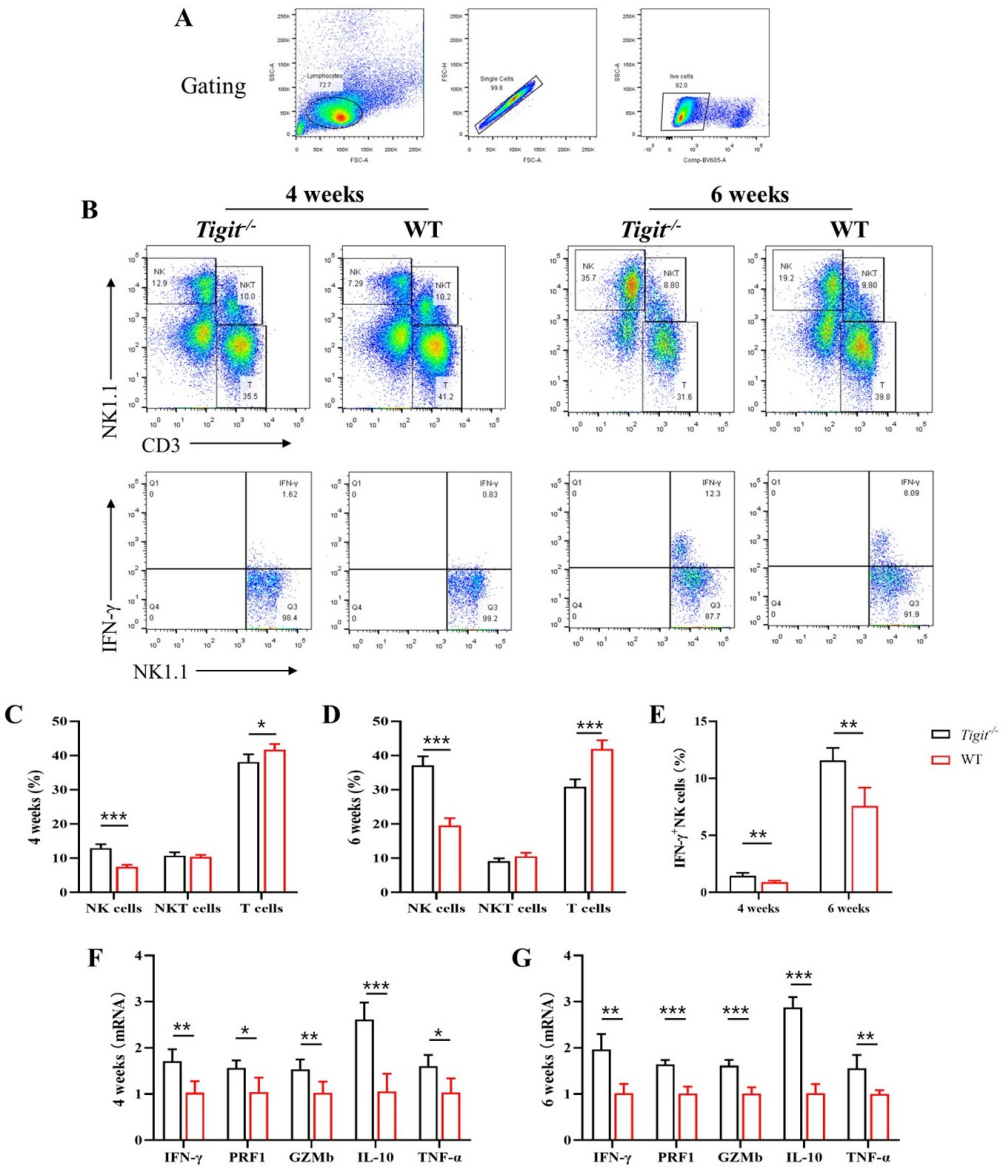
*Tigit* knockout enhanced natural killer (NK) cell proportion and activity. (A) Gating strategies for lymphocytes, single cells, and viable cells in hepatic non-parenchymal cells. (B) Flow cytometry scatter plots indicating the proportions of NK cells, natural killer T (NKT) cells, T cells, and IFN-γ^+^ NK cells at 4 and 6 weeks post infection in *Tigit*^*-/-*^ and WT mice. (C-D) Histogram of the proportions of hepatic NK, NKT, and T cells in *Tigit*^*-/-*^ and WT mice at 4 and 6 weeks post infection. (E) Histogram of the IFN-γ^+^ NK cell population in *Tigit*^*-/-*^ and WT mice at 4 and 6 weeks post infection. (F- G) Relative mRNA transcript levels of *Ifn-γ*, *Prf1*, *Gzmb*, *Il-10*, and *Tnf-α* in hepatic NK cells derived from *Tigit*^*-/-*^ and WT mice at 4 and 6 weeks post infection. *P < 0.05, **P < 0.01, ***P < 0.001.

Schistosomiasis mouse models were established by infecting WT and *Tigit*^*-/-*^ C57BL/6 mice with *S*. *japonicum* cercariae to assess the effect of *Tigit* on the proportion and function of NK cells. The results demonstrated that at 4 and 6 weeks post infection, the proportion of hepatic NK cells was higher in *Tigit*^*-/-*^ mice than in WT mice (Fig 4A–4D). In contrast, the proportion of hepatic T cells was lower in *Tigit*^*-/-*^ mice than in WT mice (Fig 4C and 4D). Flow cytometry (Fig 4E) and RT-qPCR (Fig 4F and 4G) showed that the levels of effector molecules in hepatic NK cells, such as IFN-γ, Prf1, Gzmb, IL-10, and TNF-α, were higher in *Tigit*^*-/-*^ mice than in WT mice at 4 and 6 weeks post infection. The findings indicate that at 4 and 6 weeks post infection, *Tigit* knockout increased the proportion of NK cells and promoted the secretion of effector molecules by hepatic NK cells.

### *Tigit* knockout reduced the degree of liver fibrosis in schistosomiasis

**Fig 5:**
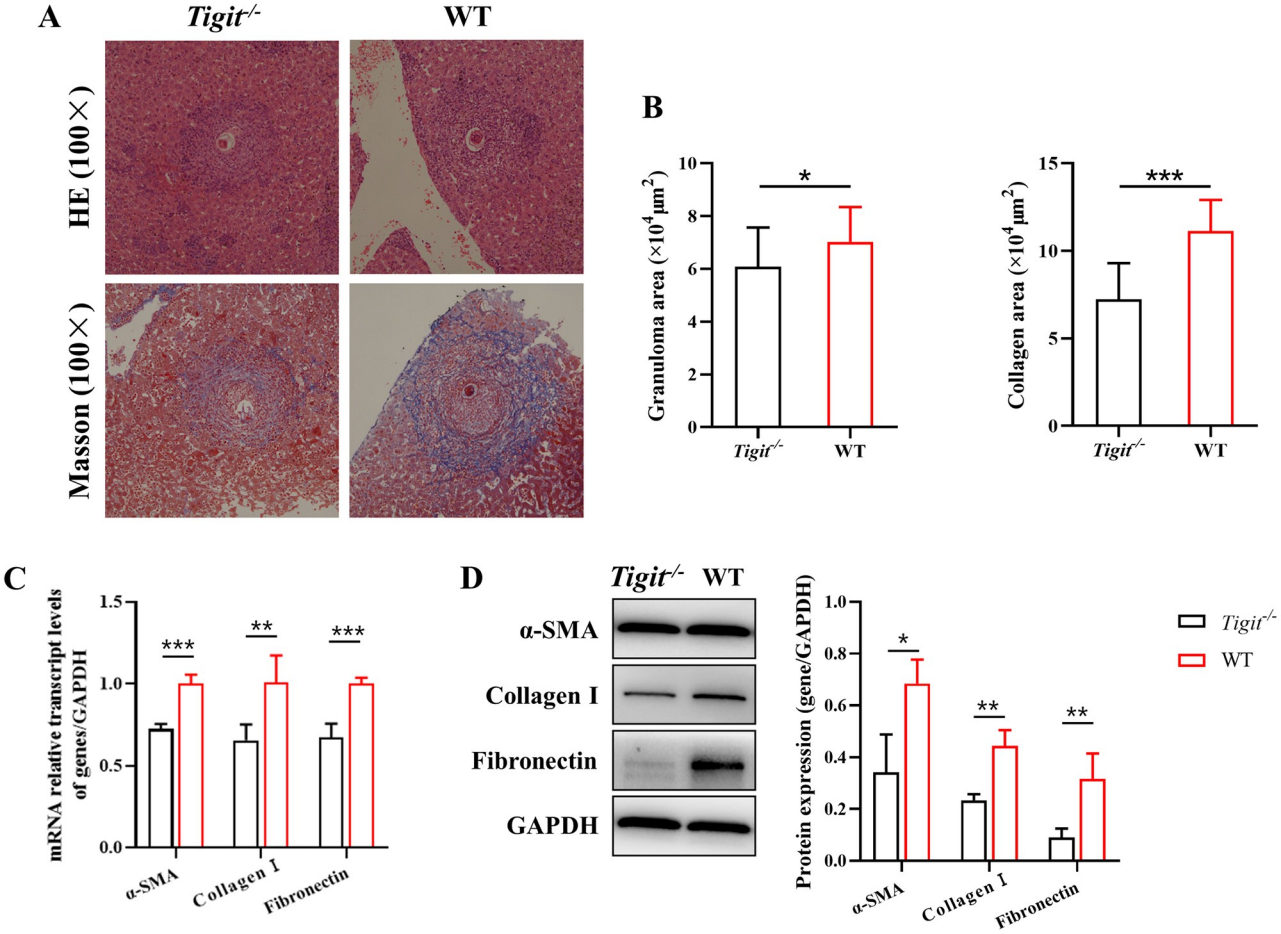
*Tigit* knockout reduces the degree of liver fibrosis in schistosomiasis. (A) Liver tissues of *Tigit*^*-/-*^ and wild-type (WT) mice were stained with hematoxylin-eosin (HE) and Masson trichome at 6 weeks post infection. (B) Liver granuloma and collagen area at 6 weeks post infection in *Tigit*^*-/-*^ and WT mice. (C) Relative mRNA transcript levels of α-SMA, collagen-I, and fibronectin in *Tigit*^*-/-*^ and WT mice at 6 weeks post infection. (D) Protein expression levels of α-SMA, collagen-I, and fibronectin in *Tigit*^*-/-*^ and WT mice at 6 weeks post infection. All data are presented as mean ± standard deviation of at least three independent experiments. *P < 0.05, **P < 0.01, ***P < 0.001.

The schistosomiasis-induced liver fibrosis of *Tigit*^*-/-*^ and WT mice was assessed 6 weeks post infection. Hematoxylin–eosin (HE) and Masson staining showed (Fig 5A and 5B) that at 6 weeks post infection, the granulomas and collagen deposition in the liver were significantly smaller in *Tigit*^*-/-*^ mice than in WT mice. RT-qPCR (Fig 5C) and western blot (Fig 5D) showed that the relative expression levels of α-SMA, collagen I, and fibronectin in the liver at 6 weeks post infection were significantly lower in *Tigit*^*-/-*^ mice than in WT mice. The changes in the levels of Th1- and Th2-type cytokines in the liver of *Tigit*^-/-^ mice and WT mice after infection were detected by qPCR (S1 Fig). The levels of Th1-type cytokines (IFN-γ, TNF-α) and immune inhibitory cytokines (IL-10) in *Tigit*^-/-^ mice were higher than those in WT mice at 4 and 6 weeks after infection. In contrast, levels of Th2 type cytokines (IL-4 and IL-13) in *Tigit*^-/-^ mice were lower than those in WT mice at 4 weeks post infection. In contrast, no significant difference in the levels of Th2-type cytokines was observed between *Tigit*^-/-^ and WT mice at 6 weeks post infection. The spleen index was lower in *Tigit*^-/-^ mice than in WT mice at 4 and 6 weeks after infection (S2 Fig). The findings indicate that the immune response of *Tigit*^-/-^ mice was weaker than that of WT mice.

### *Tigit*^-/-^ natural killer cells regulated schistosomiasis-induced liver fibrosis

**Fig 6:**
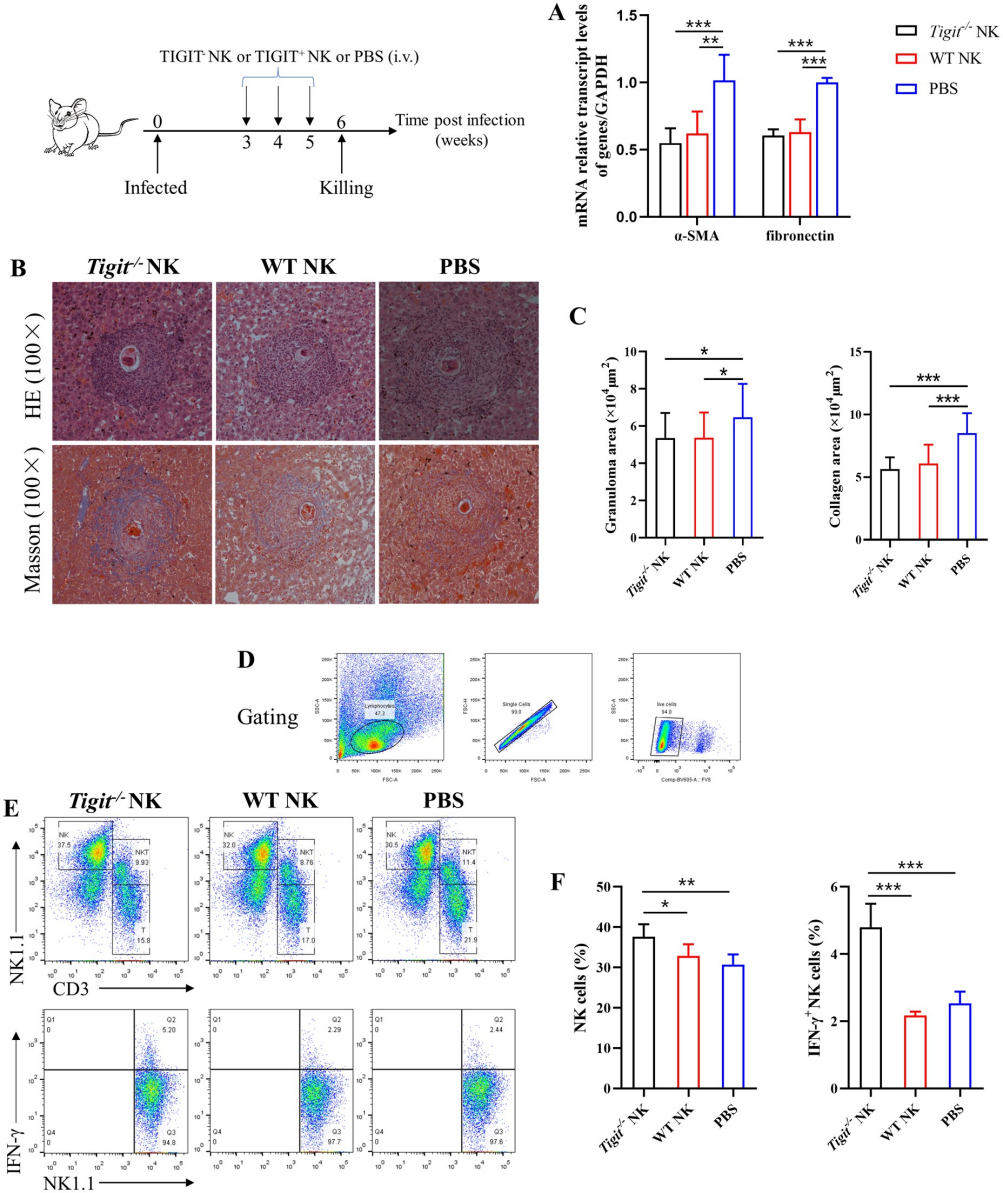
*Tigit* regulates the natural killer (NK) cell population and function of schistosomiasis-induced liver fibrosis. (A) Relative mRNA transcript levels of α-SMA and fibronectin in the *Tigit*^*-/-*^ NK cell and WT NK cell adoptive transfer groups and control (phosphate-buffered saline [PBS]) group at 6 weeks post infection. (B) Hematoxylin-eosin (HE) and Masson staining of liver tissues in the *Tigit*^*-/-*^ NK cell group, WT NK cell group, and control group at 6 weeks post infection. (C) At 6 weeks post infection, areas of liver granulomas and collagen deposition in the *Tigit*^*-/-*^ NK cell group, WT NK cell group, and control group. (D) Circle gating strategy for lymphocytes, single cells, and live cells in hepatic non-parenchymal cells. (E and F) Flow cytometry results and bar graphs of the proportion of hepatic NK cells and IFN-γ^+^ NK cells in each treatment group. All data are presented as mean ± standard deviation at least three independent experiments. *P < 0.05, **P < 0.01, ***P < 0.001.

Spleen-derived NK cells from *Tigit*^*-/-*^ and WT mice were adoptively transferred into WT mice infected with *S*. *japonicum*. We assessed the effect of the adoptive transfer of NK cells on the proportion and function of NK cells and the degree of liver fibrosis RT-qPCR showed that the relative expression levels of α-SMA and fibronectin were significantly lower in both *Tigit*^*-/-*^ and WT NK cell adoptive transfer groups than in the control group at 6 weeks post infection. However, there were no significant differences in α-SMA and fibronectin expression levels between *Tigit*^-/-^ and WT NK cell adoptive transfer groups (Fig 6A). The HE and Masson staining (Fig 6B and 6C) showed that the areas of liver granulomas and fibrosis were significantly lower in both *Tigit*^-/-^ and WT NK cell adoptive transfer groups than in the control group at 6 weeks post infection. There was no statistically significant difference between the *Tigit*^-/-^ and WT NK cell adoptive transfer groups. At 6 weeks post infection, the proportion of hepatic NK cells and IFN-γ^+^ NK cells was significantly higher in the *Tigit*^-/-^ NK cell adoptive transfer group than in the WT NK cell adoptive transfer group and control group (Fig 6D–6F). Together, the gene and protein expression results indicate that *Tigit* knockout has a regulatory effect on NK cell abundance and function.

## Discussion

Schistosomiasis affects more than 250 million people worldwide, accounting for an estimated loss of 1.9 million disability-adjusted life years (DALYs), which poses a serious threat to human health [16,17]. Three species of schistosomes, *Schistosoma haematobium*, *S*. *mansoni*, and *S*. *japonicum*, are primarily responsible for human infections. In China, *S*. *japonicum* is mainly prevalent [18]. Although the prevalence of *S*. *japonicum* in China has significantly reduced, Hu et al. reported that patients with schistosomiasis exhibit liver fibrosis [19]. In schistosomiasis, liver pathology is critically regulated by the Th1/Th2 responses. Animal studies suggest a moderate Th1 response to parasite antigens during the acute stage, whereas egg-derived antigens induce a sustained and dominant Th2 response that mediates granuloma formation and liver fibrosis [20]. Consequently, antifibrotic treatment for Th1 response is required to prevent the progression of liver fibrosis in schistosomiasis populations.

NK cells play an important role in regulating liver fibrosis. The binding of NK cell receptors such as NKG2D and NKp46 with the corresponding ligands on the surface of HSCs stimulates the antifibrotic activity of NK cells [9,21,22]. NK cells induce HSC apoptosis not only through direct contact between cells but also through the production of IFN-γ [10] or expression of apoptosis-related ligands such as FASL and TRAIL [23], or they directly kill HSCs by releasing Prf and Gzmb [24]. A previous study found that in mice infected with *S*. *japonicum*, hepatic NK cells inhibited liver fibrosis in the early stages of liver fibrosis development. NK cells are enriched in the liver and exhibit distinct phenotypes and functions [25].

In the present study, the KEGG pathways significantly enriched in the common DEGs observed at 4 and 6 weeks post infection were mostly associated with metabolism and inflammation regulatory pathways. Because the mice were in the acute inflammatory response phase at these time points after the *S*. *japonicum* infection, NK cells were activated and involved in the regulatory response. The KEGG pathways significantly enriched in the common DEGs observed at 8 and 6 weeks post infection were associated with inflammation and Th cell differentiation, particularly Th17 cell differentiation. Marina et al. reported that NK cells regulated the T-bet^+^ Th17 cell effector function through the NKG2D receptor, increasing the production of proinflammatory cytokines [26].

In our previous study, the expression of IFN-γ, Prf1, and Gzmb in NK cells increased between 2 and 4 weeks post infection and decreased between 6 and 10 weeks post infection [15]. Transcriptome sequencing in the present study revealed that the expression levels of *Ifn-γ*, *Prf1*, and *Gzmb* were significantly lower 6 weeks post infection than 4 weeks post infection, indicating that NK cells were in an inhibited state at 6 weeks post infection. This finding is consistent with previous results [15]. NK cell function is regulated by a combination of surface inhibitory and activating receptors that recognize their respective ligands on target cells or antigen-presenting cells. Targeting these receptors may regulate NK cell function or even affect the progression of liver fibrosis [27]. TIGIT expression on NK cells in uninfected mice was low. However, with the progression of *S*. *japonicum* infection, TIGIT expression increased significantly, indicating that the TIGIT receptor plays a crucial role in inhibiting NK function in liver fibrosis induced by *S*. *japonicum* infection.

TIGIT, an inhibitory receptor expressed on lymphocytes, has recently been considered an important target for cancer immunotherapy [28]. TIGIT (also known as WUCAM, Vstm3, or VSIG9) is a receptor of the Ig superfamily that is crucial to the immune response [29]. In humans and mice, TIGIT is expressed in NK cells and T cells [30]. TIGIT expression is typically low in resting cells but is upregulated in activated T cells and NK cells [31]. TIGIT acts as a ligand for CD155, indirectly by interfering with CD226 co-stimulation or directly by delivering inhibitory signals to effector cells [32]. Blockade of TIGIT prevented NK cell depletion and promoted NK cell-mediated killing [33]. TIGIT interaction with CD155 expressed on antigen-presenting cells inhibited NK cell cytotoxicity and IFN-γ production, which could be reversed by blocking TIGIT with anti-TIGIT antibodies [34]. Thus, *Tigit* knockout or TIGIT blockade with the anti-TIGIT monoclonal antibody both increased IFN-γ production and enhanced the killing effect of NK cells.

In the present study, Tigit knockout resulted in a significant increase in the levels of effector molecules secreted in NK cells and induced apoptosis in a higher proportion of HSCs compared with the WT group, suggesting that TIGIT inhibited the killing function of NK cells. The degree of liver fibrosis in *Tigit*^-/-^ mice infected with *S*. *japonicum* was significantly lower than that in infected WT mice, which indicated that *Tigit* knockout significantly inhibited liver fibrosis induced by *S*. *japonicum* egg deposition. The degree of liver fibrosis in mice adoptively transferred with activated *Tigit*^-/-^ NK cells and WT NK cells through tail vein injection was lower than that in control mice.

The present study has some limitations. First, no significant difference was observed between mice adoptively transferred with *Tigit*^-/-^ NK cells and WT NK cells. This finding may be attributed to the insufficient number of NK cells transferred; only 1× 10^5^ NK cells isolated from the spleen of two mice were transferred to one recipient mouse. The number of transferred cells needs to be increased in future studies. Second, we only explored the anti-fibrotic effect of NK cells. Other cells such as CD8^+^ T cells also play a similar role *in vivo* [35]. Tigit receptors are expressed on both NK cells and T cells. Therefore, *Tigit* knockout mice showed significantly less fibrosis than WT mice, likely owing to the contribution of NK cells and CD8^+^ T cells.

NK cells represent a mixed cell population, and the subpopulation with a killing function needs to be further investigated. Wijaya [36] reported that in chronic hepatitis B infection, KLRG1^+^ NK cells exert antifibrotic effects by releasing highly cytotoxic proteins and IFN-γ. Eisenhardt [37] demonstrated that the CXCR3^+^CD56^bright^ phenotype is a subgroup of NK cells with antifibrotic potential demonstrating a strong killing effect on HSCs and abnormal activity in hepatitis C. Choi [38] found that the activation of mGluR5 in NK cells alleviated liver fibrosis by increasing cytotoxicity and IFN-γ production.

The killing effect of *Tigit*^-/-^ NK cells is limited. It has been reported that targeting two or more immune checkpoints such as TIGIT, PD-1, and CTLA-4 significantly alters NK cell function [39]. Yu et al. [40] found that TIGIT and Tim-3 together mediate NK cell exhaustion in HCC patients, characterized by decreased expression of cytokines (IFN-γ and TNF-α) and cytotoxicity (CD107a). Xu et al. [41] concluded that the synergistic blockade of TIGIT and CD112R in NK cells significantly increased the expression of IFN-γ and CD107a and enhanced the killing effect on tumor cells compared with single receptor blockade.

In the present study, we identified that TIGIT inhibited NK cell function and reduced the degree of schistosomiasis-induced liver fibrosis. The study provides a foundation for further exploration of the mechanism of NK cell activation in schistosomiasis-induced liver fibrosis and a novel target for immunotherapy of liver fibrosis.

## Materials and methods

### Ethics statement

All experiments involving C57BL/6 mice were performed according to the recommendations of the Laboratory of Animal Welfare and Ethics Committee (LAWEC) of China. The study protocol was approved by the LAWEC Committee of the National Institute of Parasitic Diseases, Chinese Center for Disease Control and Prevention (Chinese Center for Tropical Diseases Research)(IPD-2020-10).

### Mice, parasites, and infection

Specific-pathogen-free (SPF) female C57BL/6 mice (6–8 weeks old; body weight 20 ± 2 g) were purchased from Shanghai Jihui Laboratory Animal Co., Ltd. (Shanghai, China). *Tigit*^-/-^ mice were purchased from the Shanghai Model Organisms Center, Inc. (Shanghai, China) and housed in an SPF-grade animal room at the Institute of Parasitic Disease Prevention and Control, Chinese Center for Disease Control and Prevention. Animals were randomly allocated to certain groups before the start of the study.

*S*. *japonicum* cercariae were obtained from the National Institute of Parasitic Diseases, Chinese Center for Disease Control and Prevention (Shanghai, China). Mice were percutaneously infected by applying approximately 20 cercariae to the shaved skin of the abdomen. C57BL/6 mice were anesthetized and euthanized at 0, 2, 4, 6, and 8 weeks after infection. The spleen index was calculated in *Tigit*^-/-^ and WT mice at 4 and 6 weeks after infection according to the formula described below:

Spleenindex=mousespleenweight/mousebodyweight

### Library preparation for RNA sequencing

NK cells were isolated from non-parenchymal liver cells. For each sample, mRNA was enriched using oligo-dT magnetic beads and then fragmented in a fragmentation buffer. First-strand cDNA was synthesized from the cleaved RNA fragments using random hexamer primers. Second-strand cDNA was synthesized in a reaction mixture containing 5× first-strand buffer, deoxyribonucleotide triphosphates, RNase H, and DNA polymerase I. The double-stranded cDNA was blunted and phosphorylated at the 5′-end. The 3′-end formed a sticky end with a protruding "A" connected with a bubble-shaped linker with protruding "T" at the 3′-end. The ligation product was amplified by PCR. The amplification products were thermally denatured into single-stranded DNA and circularized with a bridge primer to obtain a single-stranded circular DNA library. We assessed the library integrity and size distribution using an Agilent 2100 Bioanalyzer (Agilent Technologies, Santa Clara, CA, USA). The cDNA library was sequenced on a paired-end flow cell using the BGISEQ-50 platform (BGI, Shenzhen, China).

### Analysis of sequencing data

First, we eliminated adapter-containing reads, low-quality reads, and poly-N- containing reads from the raw data to obtain clean data using the filtering software SOAPnuke (https://github.com/BGI-flexlab/SOAPnuke). Next, we estimated the GC content, Q30, and sequence duplication level. Then, we mapped the high-quality clean reads aligned to the reference genome sequence utilizing HISAT [42]. The gene expression levels were calculated as fragments per kilobase of exon per million mapped fragments (FPKM) of different samples, using the following formula: FPKM = cDNA fragments/mapped fragments (millions) × transcript length (kb).

### Differentially expressed genes and enrichment analysis

DEGs between the two groups were identified using the DESeq package in R [43]. DESeq supplies statistical routines for confirming the differential expression in digital gene expression data utilizing a model based on the negative binomial distribution. Genes with an expression fold change (FC) ≥2 and adjusted *P*-value ≤0.001, as determined by DESeq, were considered DEGs. The DEGs were analyzed using the phyper function in R software using the KEGG pathway database (http://www.kegg.jp/kegg/pathway.html) to obtain GO annotations and KEGG pathway data. FDR correction was used to determine the significantly enriched GO categories and KEGG pathways (corrected *P*-values ≤ 0.05).

### Cell isolation

C57BL/6 mice were sacrificed via cervical dislocation after anesthesia. Mouse liver was perfused using 1× Dulbecco’s phosphate-buffered saline (DPBS) via the portal vein to remove the remaining blood from the tissue. The liver was then removed and washed in DPBS. The tissue was quickly minced with scissors and dissociated into single-cell suspensions using an Ultra Turrax tube disperser (IKA, Königswinter, Germany). Cells were separated by differential gradient centrifugation. The supernatant and lipid layer was discarded, and the cells were washed twice with DPBS. The red cells were lysed using BD Pharm Lyse Lysing Buffer (Becton Dickinson and Company, Franklin Lakes, NJ, USA) to obtain hepatic non-parenchymal cells.

The concentration of hepatic non-parenchymal cells was adjusted to 1 × 10^7^ cells. We used the NK Cell Isolation Kit (Miltenyi Biotec, Auburn, CA, USA) to isolate NK cells. The cell suspension was centrifuged, and the supernatant was completely removed. The cell pellet was resuspended in 90 μl of buffer (phosphate-buffered saline, pH 7.2; 0.025% bovine serum albumin [BSA]; and 0.1mM ethylenediaminetetraacetic acid [EDTA]). Then, 10 μl of CD49b (DX5) MicroBeads per 10^7^ total cells were added, and the mixture was incubated in a 4°C refrigerator for 15 min. The cells were washed by adding 1−2 ml of buffer and centrifuged at 300 × *g* for 10 min; the supernatant was completely removed using a pipette. The cell suspension was applied to the column. The fraction of magnetically labeled cells, representing the enriched NK cells, was collected.

### Natural killer cell co-culture with hepatic stellate cells and apoptosis assay

mHSCs were cultured at 37°C with 5% CO_2_ in Dulbecco’s Modified Eagle Medium containing 10% FBS, 100 U/ml penicillin, and 100 μg/ml streptomycin. The cells were harvested and stained with propidium iodide and fluorescein isothiocyanate-conjugated annexin V (BD Biosciences) to detect apoptosis, according to the manufacturer’s instructions. Target cells alone were used as controls. Hepatic NK cells from *Tigit*^-/-^ and WT mice were co-cultured with mHSCs at an effector-to-target ratio of 5:1 for 24 or 48 h, and the percentage of apoptotic mHSCs was calculated.

### Flow cytometry

The concentration of hepatic non-parenchymal cells was adjusted to 1 × 10^6^/ml using fluorescence-activated cell sorting (FACS) buffer (2% BSA and 2 mM EDTA in DPBS). The following antibodies were used in our experiments: Fixable Viability Stain 575V and CD3-APC-CY7, NK1.1-BV421, TIGIT-PE, CD3-BUV395, NK1.1-PE-CY7, and IFN-γ-APC. These antibodies were purchased from BD Biosciences (San Jose, CA, USA). NK cells were defined as CD3^-^NK1.1^+^ Cells were stained with different combinations of antibodies for 30 min at room temperature (24–26°C) in the dark, followed by one wash with FACS buffer. All experiments were performed using a BD FACS Verse flow cytometer (BD Biosciences). Data were analyzed using FlowJo 10 software (TreeStar Inc., Ashland, OR, USA). The proportion of NK cells and level of IFN-γ secretion by NK cells were measured.

### Reverse transcription-quantitative PCR

Total RNA was extracted from mouse livers, and cDNA was synthesized. Total RNA from NK cells was extracted using the TRIzol method. Complementary DNA (cDNA) was synthesized using 1 μg of total RNA with a Prime Script RT Master Mix (Takara, Shiga). RT- qPCR was used to determine the expression of genes, including those in liver samples (*α-Sma*, collagen I, fibronectin) and NK cell samples (*Tigit*, *Ifn-γ*, *Gzmb*, *Prf1*, *Tnf-α*) using Fast SYBR Green master Mix (Bio-Rad, Hercules, CA, USA). Primer sequences were shown in Table 1.

**Table 1:** Primer sequences for reverse transcription-quantitative PCR.

Gene	Forward (5′–3′)	Reverse (5′–3′)
GAPDH	GTGTTCCTACCCCCAATGTG	GTCATTGAGAGCAATGCCAG
α-SMA	CTGGTATTGTGCTGGACTCTG	GATCTTCATGAGGTAGTCGGTG
Collagen I	CCTCAGGGTATTGCTGGACAAC	CAGAAGGACCTTGTTTGCCAGG
Fibronectin	GGTCCTCTCCTTCCATCTCCTTAC	GGACCCCTGAGCATCTTGAGTG
TIGIT	CATACGTATCCTGGTGGGATTTAC	TACAGTCACTCCTGTGACCATTAAG
IFN-γ	CAGCAACAGCAAGGCGAAAAAGG	TTTCCGCTTCCTGAGGCTGGAT
Gzmb	AGGCCAATGGAACACCTCTTC	GGAGAGGGCAAACTTCCATAGG
Prf1	ACACAGTAGAGTGTCGCATGTAC	GTGGAGCTGTTAAAGTTGCGGG
TNF-α	TCTTCTCATTCCTGCTTGTGGC	GGTCTGGGCCATAGAACTGATG
IL-10	CGGGAAGACAATAACTGCACCC	CGGTTAGCAGTATGTTGTCCAGC
IL-4	GAACTCTAGTGTTCTCATGGAGCTG	TCTTTCAGTGATGTGGACTTGGAC
IL-13	CCTCATGGCGCTTTTGTTGAC	TCTGGTTCTGGGTGATGTTGA

### Western blotting

Liver tissues of *Tigit*^-/-^ mice and WT mice were collected at 6 weeks post infection and lysed using radioimmunoprecipitation assay (RIPA) lysis buffer (Shanghai Epizyme Biomedical Technology Co., Ltd, China) supplemented with protease inhibitor cocktail and EDTA (Beyotime Biotechnology, China). The lysates were centrifuged at 12 000 × *g* for 10 min, and the cellular debris was discarded. The collected supernatant was diluted to different concentrations and used to determine protein concentration by the BCA method. An appropriate amount of protein loading buffer was added to the protein sample. The mixture was placed in a metal bath at 100°C for 10 min, cooled to room temperature, and immediately loaded into the sample well of a sodium dodecyl sulfate (SDS)-polyacrylamide gel (Beyotime Biotechnology, China). SDS-polyacrylamide gel electrophoresis was performed at 90 V for approximately 1 h. The proteins separated by electrophoresis were transferred to polyvinylidene fluoride (PVDF) membranes. After blocking, the membranes were incubated sequentially with primary and secondary antibodies. The following primary antibodies were used: anti-GAPDH (5174S, Cell Signaling Technology, CST, USA), anti-a-SMA (19245S, CST), anti-Col 1 a1 (bs-7158R, Bioss), and anti-fibronectin (63779s, CST). Horseradish peroxidase-conjugated anti-mouse IgG was used as the secondary antibody (7076S, CST). The PVDF membranes were washed three times with Tris-buffered saline (TBS) containing 0.5‰ Tween-20. Immunoreactive bands were visualized on digital images using a ChemiDoc MP Imaging System (Bio-Rad). The band intensities were quantified by ImageJ software.

### Histopathology of liver fibrosis

The right lobes of mouse livers were fixed in 4% paraformaldehyde. Liver tissues were dehydrated and embedded in paraffin using routine procedures. Paraffin sections (4 μm) were prepared from each liver tissue sample. The sections were stained with HE and Masson trichrome. Sections stained with HE were examined under the optical microscope at 100× magnification to observe the development and changes in *S*. *japonicum* egg granulomas. Sections stained with Masson’s trichrome, which stains collagen blue were examined to monitor changes in collagen deposition. The areas of granulomas and fibrosis surrounding single eggs were evaluated using Image J (NIH, USA).

### Isolation and adoptive transfer of natural killer cells

Mature NK (CD3^-^ NK1.1^+^) [44] cells were isolated from the spleens of WT and *Tigit*^-/-^ mice by fluorescence-activated cell sorting. The following antibodies were used for staining: FVS-BV605, CD3-APC-cy7, and NK1.1-PE. The sorted NK cells were washed and resuspended in complete medium and cultured in complete medium with IL-2 (10 ng/ml), IL-15 (10 ng/ml), and IL-18 (25 ng/ml) [45]. After 6 h, the cells were washed with a complete medium, counted, and resuspended in phosphate-buffered saline (PBS). Eighteen C57BL/6 mice were randomly divided into three groups, with six mice per group: *Tigit*^-/-^ NK cell transfer group, WT NK cell transfer group, and PBS group. Each mouse was infected with (20 ± 1) cercariae through abdominal skin. At 3 weeks post infection, the mice in the three groups were injected with *Tigit*^-/-^ NK cells, WT NK cells (2 × 10^5^ cells/200 μl per mouse), or 200 μl PBS through the tail vein; the injections were administered weekly for three consecutive weeks. At 6 weeks post infection, the mice were sacrificed and dissected.

### Statistical analysis

Data analysis was performed using GraphPad Prism Version 8 and SPSS 20.0 (IBM Corp., Armonk, NY, USA). Differences between groups were assessed using a nonparametric one-way analysis of variance. Data were presented as mean ± standard deviation. *P* <0.05 indicated a significant difference. Outliers were identified and removed.

## Supporting information

S1 Data Excel spreadsheet containing, in separate sheets, the numerical data for figure panels 1A, 1B, 1C, 1H, 1I, 1J, 2A, 2B, 2C, 3B, 4C, 4D, 4E, 4F, 4G, 5B, 5C, 5D, 6A, 6C, and 6F. (XLS)

S2 Data Original picture for the immunoblotting results is shown in Fig 5D. (PDF)

S1 Fig Changes in cytokine expression in *Tigit*^-/-^ and wild-type (WT) mouse liver tissue at (A) 4 weeks after infection and (B) 6 weeks after infection. (TIF)

S2 Fig Changes in the spleen index of *Tigit*^-/-^ and wild-type (WT) mice at 4 and 6 weeks after infection. (TIF)
