# Supplementary figures and images for: Inhibition of hepatic natural killer cell function via the TIGIT receptor in schistosomiasis-induced liver fibrosis
Source: PLoS Pathog. 2023 Mar 17;19(3):e1011242. doi: 10.1371/journal.ppat.1011242 (PMC10022799; doi:10.1371/journal.ppat.1011242)

Figure 5D

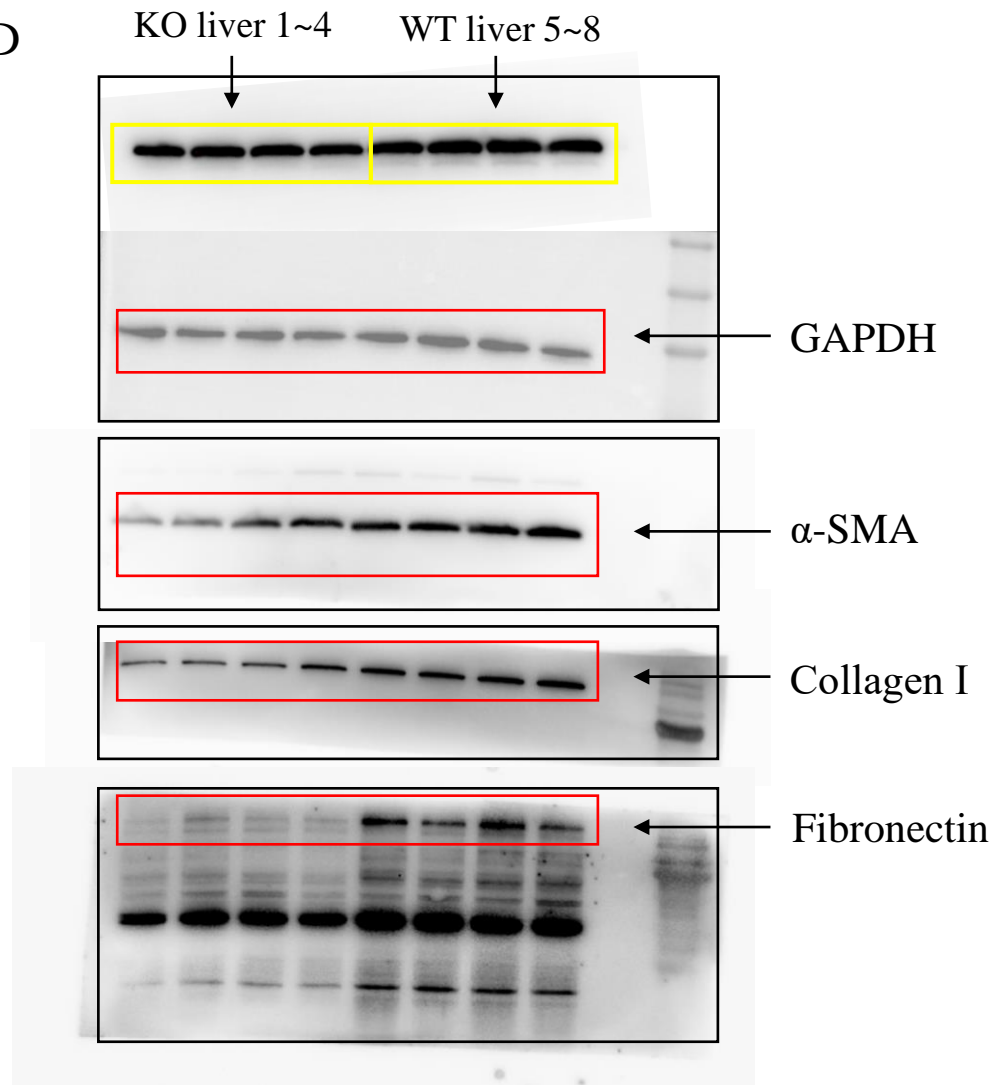

Supplement: S2 Data — (PDF) [file ppat.1011242.s002.pdf]

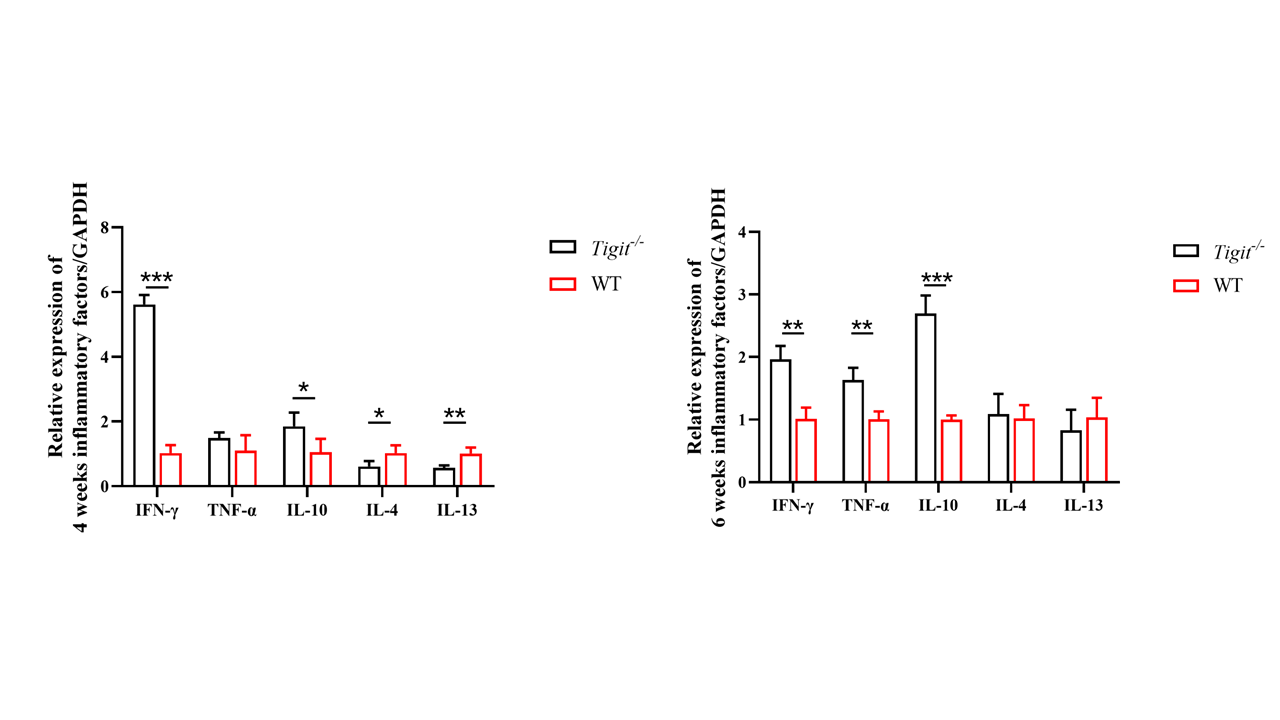

Supplement: S1 Fig — (TIF) [file ppat.1011242.s003.tif]

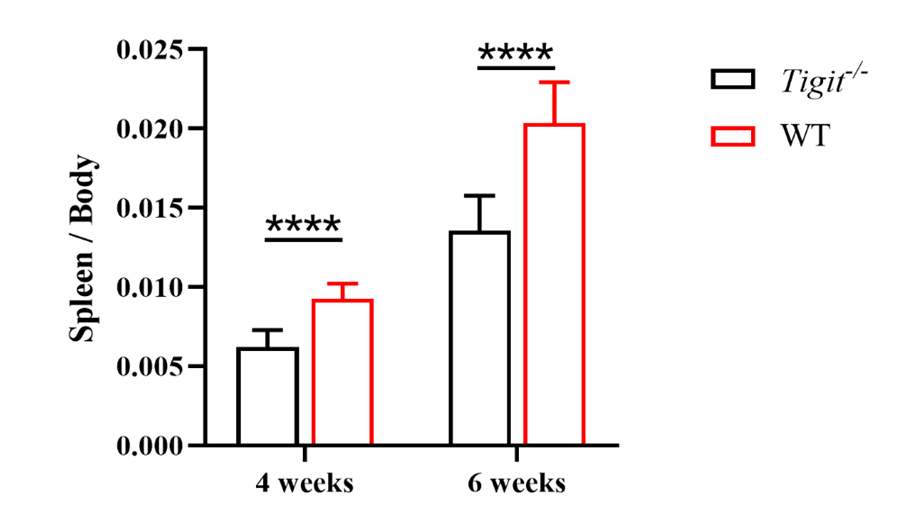

Supplement: S2 Fig — (TIF) [file ppat.1011242.s004.tif]
